# Cerebral Representation of Sound Localization Using Functional Near-Infrared Spectroscopy

**DOI:** 10.3389/fnins.2021.739706

**Published:** 2021-12-14

**Authors:** Xuexin Tian, Yimeng Liu, Zengzhi Guo, Jieqing Cai, Jie Tang, Fei Chen, Hongzheng Zhang

**Affiliations:** ^1^Department of Otolaryngology Head & Neck Surgery, Zhujiang Hospital, Southern Medical University, Guangzhou, China; ^2^Department of Electrical and Electronic Engineering, Southern University of Science and Technology, Shenzhen, China; ^3^Department of Physiology, School of Basic Medical Sciences, Southern Medical University, Guangzhou, China; ^4^Hearing Research Center, Southern Medical University, Guangzhou, China; ^5^Key Laboratory of Mental Health of the Ministry of Education, Southern Medical University, Guangzhou, China

**Keywords:** sound localization, functional near-infrared spectroscopy (fNIRS), spatial hearing, cerebral cortex, auditory cortex (AC), dorsolateral prefrontal cortex (dlPFC)

## Abstract

Sound localization is an essential part of auditory processing. However, the cortical representation of identifying the direction of sound sources presented in the sound field using functional near-infrared spectroscopy (fNIRS) is currently unknown. Therefore, in this study, we used fNIRS to investigate the cerebral representation of different sound sources. Twenty-five normal-hearing subjects (aged 26 ± 2.7, male 11, female 14) were included and actively took part in a block design task. The test setup for sound localization was composed of a seven-speaker array spanning a horizontal arc of 180° in front of the participants. Pink noise bursts with two intensity levels (48 dB/58 dB) were randomly applied *via* five loudspeakers (–90°/–30°/–0°/+30°/+90°). Sound localization task performances were collected, and simultaneous signals from auditory processing cortical fields were recorded for analysis by using a support vector machine (SVM). The results showed a classification accuracy of 73.60, 75.60, and 77.40% on average at –90°/0°, 0°/+90°, and –90°/+90° with high intensity, and 70.60, 73.6, and 78.6% with low intensity. The increase of oxyhemoglobin was observed in the bilateral non-primary auditory cortex (AC) and dorsolateral prefrontal cortex (dlPFC). In conclusion, the oxyhemoglobin (oxy-Hb) response showed different neural activity patterns between the lateral and front sources in the AC and dlPFC. Our results may serve as a basic contribution for further research on the use of fNIRS in spatial auditory studies.

## Introduction

Auditory perception is one of the most important sensory modalities in creatures. There are multiple types of information presented in sounds. Identifying the source of the sound makes wild animals aware of the danger or its prey and is important in communicative interactions in human society. For decades, auditory neuroscientists have examined the neuronal mechanisms underlying spatial hearing (Middlebrooks and Green, 1991; Skottun, 1998; Grothe et al., 2010). For mammals, the localization and identification of sounds are constructed from the precise relative intensity and timing between the two ears [two binaural cues mostly play roles in the horizontal plane: interaural time difference (ITD) and interaural level difference (ILD)] as well as from patterns of frequencies mapped at the two ears (play roles mostly in the vertical plane) (Middlebrooks and Green, 1991). In addition to the acoustic features, scientists found that the behavioral state of a listener (like task performance and attention) affects neuronal spatial selectivity (Harrington et al., 2008; van der Heijden et al., 2018). Taken together, humans integrate input from the ears and cognitive processes to derive the location of sound sources (Nothwang, 2016; Zhang and Liu, 2019). However, the neural encoding of sound locations and especially the processing of sound sources in the cortex remains a matter of ongoing discussion, and there are still divergent views (Ahveninen et al., 2014).

Electrophysiological research in non-human primates and non-invasive research in humans have provided evidence from a neuroanatomical and functional perspective for acoustic spatial neuron encoding. Regarding the insights into the cortical encoding, evidence for a broader dichotomy between the anterior “what” vs. posterior “where” pathways of the non-primary auditory cortex (AC) aggregates from human neuroimaging studies (Ahveninen et al., 2006; Barrett and Hall, 2006). The dorsal “where” pathway views sound localization as a higher-order sound attribute in higher-level areas including inferior parietal lobule, premotor cortex, dorsolateral prefrontal cortex (dlPFC), and inferior frontal cortex (Rauschecker, 2018; Czoschke et al., 2021). Several published studies have mentioned that the planum temporale (PT) plays an essential role in mediating human horizontal sound localization. Functional MRI (fMRI) research showed that the sound location processing activates the posterior superior temporal gyrus (pSTG) and the inferior parietal cortex (Deouell et al., 2007; van der Zwaag et al., 2011). However, studies demonstrated that goal-oriented sound localization can induce adaptive changes in spectrotemporal tuning in the “dorsal” pathway areas [especially in the primary auditory cortex (PAC)], which can facilitate target detection (Atiani et al., 2009; Lee and Middlebrooks, 2013). fMRI studies reported that the dlPFC might be the source of origin of the top–down modulations that translate sensory representations into task-based representations (Jiang et al., 2018). These findings might suggest that the cortical encoding of sound localization involves recurrent and dynamic processing in PAC and higher-level areas and highlight the need for cortical representation of sound localization in spatial auditory networks.

Besides, there is a contralateral biased tuning of different sound sources with a different degree of bias across the cerebral hemisphere. Non-human primates’ measurements demonstrated that cortical spatial tuning is generally broad and predominantly contralateral (Ortiz-Rios et al., 2017). Similar spatial tuning properties have been observed in fMRI studies (Derey et al., 2016; McLaughlin et al., 2016; Higgins et al., 2017). However, inconsistent patterns were reported in human neuroimaging studies. Some electroencephalogram (EEG) and fMRI measures show that the left hemisphere (LH) responds maximally to the contralateral sound source direction and that the right hemisphere (RH) responds more equally to both the contralateral and ipsilateral sounds (Briley et al., 2013; Higgins et al., 2017). Some magnetoencephalography (EMG) studies have shown more activities in RH than LH (Johnson and Hautus, 2010; Salminen et al., 2010). Further measurements using a new image technology are needed to reveal the brain asymmetry in neural sound location encoding.

The development of functional near-infrared spectroscopy (fNIRS) has recently advanced imaging studies in acoustic and audiology, overcoming interference issues in EEG and fMRI. There is an increased oxygen requirement in the brain regions responsible for the specific functions when people are performing the relevant activity. fNIRS is an optical imaging modality that assesses brain hemodynamic responses by its inexpensiveness, safety, non-invasion, and 1–2-cm spatial resolution. This technique is designed to detect changes in the concentration of oxygenated and deoxygenated hemoglobin molecules in the blood (Leon-Carrion and Leon-Dominguez, 2012). Studies have shown that neural activity and the hemodynamic response maintain a linear relationship (Arthurs and Boniface, 2003), and the NIR signal maintains a strong correlation with PET measures of changes in regional cerebral blood flow (rCBF) and the fMRI blood oxygen level-dependent (BOLD) signal (Toronov et al., 2003; Huppert et al., 2006), suggesting that fNIRS is an effective method for assessing cerebral activity. Compared with imaging devices, such as EEG, MEG, and fMRI (Coffey et al., 2016; Dalenberg et al., 2018), fNIRS has no ill-posed inverse problem in EEG and MEG (Helmholtz, 1853) and less interference from the external environment. Whereas the spatial resolution determines anatomical details, the temporal resolution determines the precision in which we can investigate successive neuronal events. With a better spatial resolution than EEG and a similar temporal resolution of fMRI, fNIRS is a relatively good measurement of neuronal activity. In addition, fNIRS is allowable for electrical artifact and ferromagnetic component features, which suggests that fNIRS is a potential tool for the study of auditory perception in special populations.

In fNIRS studies, the existing literature on spatial auditory perception is limited and focuses mainly on speech perception, sound intensity and loudness, and the cross-modal cortex with audiovisual stimulation. For sound intensity, several new fNIRS studies were performed by Chen et al. (2015), Bauernfeind et al. (2018), and Weder et al. (2018, 2020). Those studies found evidence of a linear correlation of the hemodynamic responses with perceived loudness rather than sound intensity in the bilateral superior temporal gyrus (STG). Moreover, no interhemispheric differences are seen in the STG bilaterally. Brain asymmetry was also reported in fNIRS studies. A recent study of dichotic listening suggested that a stronger RH activity in the right prefrontal region can be observed during focused attention tasks (Eskicioglu et al., 2019). However, they neglected the effect of sound source orientation in the cortical representation. This remains the question, what is the cortical representation of a simple spatial sound source detected with fNIRS?

To our knowledge, there are no studies examining the cerebral representation in the prefrontal and auditory cortices during sound localization tasks *via* fNIRS. As fNIRS does not share the issue mentioned in EEG (ill-posed inverse problem) and fMRI (intrinsic noise), it may yield a new understanding of the cerebral cortex-modulated process and brain asymmetry in sound localization. Since localization acuity is higher for broadband than for narrowband sounds and the neural sound location encoding was influenced by the attention of listening (Butler, 1986), here, we presented pink noise bursts with different sound intensities and sources randomly in blocks of a run, allowing participants to attend the sound localization task and avoid speech understanding.

The aims of this study included the following two aspects: (1) does fNIRS detect differences in cortical representations of human attention to different sound source directions between −90°, 0°, and +90°, and if so, (2) are there differences in cortical representations for sound source orientations between −30°, 0°, and +30°? We hypothesized that our spatial stimulus presentation could result in different cerebral representations in both AC and the prefrontal cortex, showing an asymmetric bilateral cortical activation pattern.

## Materials and Methods

### Participants

**FIGURE 1:**
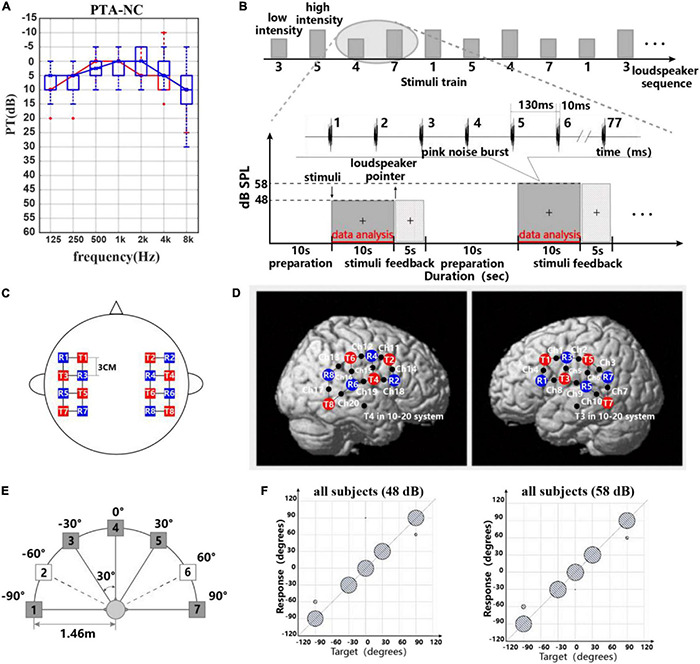
**(A)** Participants’ pure tone threshold information. **(B)** Experimental paradigm and stimulus waveform. Block design used for recording task-related hemodynamic responses: five speakers and two different intensity levels were presented in order randomly. Stimulus waveform representations of pink noise bursts. **(C,D)** Probe layout of the eight sources and eight detectors. **(C)** Placement of the fNIRS optodes (red squares are sources, blue squares are detectors, and black points on lines are channels). **(D)** Optode arrangement in both hemispheres. **(E)** Schematic representation of the seven-loudspeaker system. Loudspeaker 1 and loudspeaker 7 were placed 90° to the left and right of the straight-ahead (0°) position. Loudspeakers 2–6 were placed 30° apart between loudspeakers 1 and 7. Filled symbols indicate “active” loudspeakers; open symbols indicate “dummy” loudspeakers. **(F)** Scatter plots for sound source identification with a five-active-loudspeaker array of all subjects in 48 and 58 dB.

Twenty-five normal-hearing participants [subject1–subject25 (S1–S25), 11 males and 14 females, all right-handed, all native speakers of Chinese, ages 26.0 ± 2.7 years] took part in this study. This study was approved by the Human Subjects Committee of the Southern Medical University. All individuals were paid an hourly wage for their participation and gave written informed consent prior to the beginning of testing. Otoscopy and acoustic audiometry were conducted with each subject to determine eligibility in this study. Pure tone audiometry showed no significant difference in the hearing thresholds at frequencies 125–8,000 Hz between left (as shown in Figure 1A).

### Materials and Experiment Paradigm

#### Apparatus

The sound localization experiments were carried out in a completely darkened anechoic chamber (dimensions L × W × H = 3.3 × 3.5 × 2.5 m^3^) in which the apparatus was installed. Seven loudspeakers (Genelec 8010, Genelec Oy, Iisalmi, Finland, matched within 2 dB at 74–20,000 Hz) were positioned in a horizontal arc with a radius of *r* = 1.46 m at ear level of the subject. The speakers spanned an angle of −90° left to +90° right with a spacing of 30°. Since more sound source directions could increase the test duration and thus cause the subject fatigue, only five of seven loudspeakers (all speakers were real and available) were used for sound presentation in this experiment (Godar and Litovsky, 2010; Zaleski-King et al., 2019). A schematic diagram of the loudspeaker arrangement is shown in Figure 1E. The frequency response of each loudspeaker was individually calibrated using our experiment stimuli (seen in Figure 1B) in ±1 dB at the subject’s head position using an integrating–averaging sound level meter (Xingqiu, HS5670A). Hardware including an eight-channel Yamaha Ro8-D in conjunction with a PC host and software including dante virtual soundcard, dante controller, and MATLAB (MathWorks 2020a, United States) was responsible for stimulus presentation.

#### Experiment Paradigm and Stimuli

In the behavioral and fNIRS part, the participant was seated facing the front loudspeaker at a distance of approximately 1.46 m and was instructed to calm down and not move their body. A computer monitor placed underneath the front loudspeaker was used as part of the computerized experimental paradigm. A “+” was placed in front of the participant, and the participant was instructed to maintain eye contact with the “+” for the duration of the test. Figure 1B shows the experimental paradigm (Moghimi et al., 2012; Weder et al., 2020). At the beginning of the experiment, a preparation time of 10 s was given to the participants. Each 10-s stimulus consists of 77 pink noise bursts each with a duration of 10 ms and with a 120-ms inter-burst gap. The stimuli varied in intensity (low intensity with 48 dB SPL, high intensity with 58 dB SPL) and sound location (loudspeakers 1, 3, 4, 5, 7) (Grieco-Calub and Litovsky, 2010; Weder et al., 2018). In preliminary studies, some researchers used pink noise bursts or broadband noise bursts as a stimulus signal for acoustic source localization (Ching et al., 2005; Grantham et al., 2007; Veugen et al., 2017). The reason for using broadband noise bursts was to activate broad cortical auditory areas. Besides, compared to speech sounds, pink noise is a simpler acoustic stimulus and does not affect the cortical representation of direction recognition due to speech understanding.

During presentation of the sound for 10 s, they were asked to concentrate on the sound location internally without a head movement. Each participant was asked to point to the perceived direction of the sound source at the end of a stimulus. A surveillance camera in the anechoic chamber was used to record the feedback of the subjects. After a 10-s break, the same procedure was repeated. Each of 5 different sound locations * 2 intensity was repeated 10 times and was presented randomly during the localization test. In total, the whole test lasted for approximately 40 min. Feedback was not provided. The subject was unaware that only five of the loudspeakers were used, so that valid responses ranged from 1 to 7. Customized software for stimulus presentation and data collection was written in MATLAB programming language. We used Psychtoolbox in MATLAB to send the trigger for stimulus marking to the NIRS system.

### Data Acquisition

During the experiments, task-related cerebral hemodynamic responses were recorded using a multichannel near-infrared spectroscopy (NIRS) imaging system (LIGHTNIRS, Shimadzu Co. Ltd., Kyoto, Japan). The change of oxyhemoglobin [oxy-Hb] and deoxyhemoglobin [deoxy-Hb] and total hemoglobin [total-Hb] was calculated using a modification of the Beer–Lambert law approach. For data recording, we parted all participants’ hair and adjusted the signal-to-noise ratio of the NIRS signals using the automatic adjustment function in the measurement software (fNIRS, Shimadzu Co. Ltd., Kyoto, Japan). The signals were digitized at 13.3 Hz, and the 16 optical fiber probes consisting of eight sources (three wavelengths each source, 780, 805, and 830 nm) and eight detectors were attached to the subject’s scalp. The probe layout resulted in 20 channels, as shown in Figures 1C,D. Source and detectors were arranged over both hemispheres with 3-cm source-detector separation for maintaining acceptable signal quality and sensing depth (Power et al., 2011). The NIRS optode configuration used in this study followed previous research, which reports the engagement of the pSTG, premotor cortex, and dlPFC in binaural sound cue tuning (McLaughlin et al., 2016).

To allow probabilistic reference to cortical areas underlying the measurement channels and enable the results comparable to results provided by similar fMRI studies. Brain surface MNI (Montreal Neurological Institute) coordinates of channel midpoints were determined and fed into the SPM anatomy toolbox to allocate them to brain areas using a 3D digitizer system (FasTrak, Shimadzu, Japan). The MNI coordinates and anatomical locations of channels and regions of interest (ROIs) are shown in Table 1 (Eickhoff et al., 2005; Tsuzuki and Dan, 2014).

**TABLE 1:** Coordinates and related Brodmann and anatomical areas (based on 25 subjects).

Hem.	ROI	ch	MNI-space				Cortical areas	Proportion
			*X*	*Y*	*Z*		BA	
Left	1	1	–60	3	39	6	Pre-motor and supplementary motor cortex	0.7964
		2	–65	–18	39			0.3576
		5	–66	–6	29			0.6310
		8	–64	6	17			0.5016
	2	3	–68	–39	30	40	Supramarginal gyrus part of Wernicke’s area	0.9527
		6	–6	–29	25			0.6075
	3	4	–69	–16	27	9	dlPFC	0.5610
	4	7	–68	–50	7	22	Superior temporal gyrus	0.5290
		10	–71	–39	2			0.5092
	5	9	–68	–16	14	42	Auditory association cortex	0.4658
Right	6	11	62	2	40	6	Pre-motor and supplementary motor cortex	0.8272
		12	67	–18	41			0.3588
		15	68	–5	31			0.7785
		18	67	4	18			0.5342
	7	13	69	–40	31	40	Supramarginal gyrus part of Wernicke’s area	0.9968
		16	71	–29	27			0.7037
	8	14	63	14	27	9	dlPFC	0.6111
	9	17	69	4	18	22	Superior temporal gyrus	0.4618
		20	72	–41	1			0.5140
	10	19	71	–17	14	42	Auditory association cortex	0.4969

*The table shows 20 channels with MNI space correspondence (x, y, z with SD) and Brodmann areas (BA). The mean MNI coordinates represent the locations of the most likely MNI coordinates for the fNIRS channel projected on the cortical surface.BA, Brodmann area; STG, superior temporal gyrus; dlPFC, dorsolateral prefrontal cortex.*

### Data Analysis

#### Behavioristics

Localization performance was determined by calculating the average root-mean-square (RMS) error in degree. For each response, the loudspeaker identified by the subject as delivering the sound was recorded, resulting in a total of 100 speaker location responses for each participant. The error for each response was subsequently converted to degrees and the RMS error for each subject in each listening condition (Zheng et al., 2015). The purpose of calculating subjects’ behavioral indicators was to assess subjects’ performance in our experimental setting and to maintain subjects’ attention during the feedback task. Therefore, we did not set groups.

A non-parametric test was calculated to examine whether there were any statistically significant differences between stimulus levels (48 dB, 58 dB).

#### Functional Near-Infrared Spectroscopy Data

The fNIRS data analysis procedure consisted of preprocessing, feature extraction, feature selection, and classification stages (for details, see Power et al., 2011; Aydin, 2020). In this study, only the [oxy-Hb] data were used for data analysis, as [oxy-Hb] is a more suitable and robust parameter that has a higher correlation with the fMRI-BOLD response to investigate cortical activity (Plichta et al., 2007). Data preprocessing and analysis were executed in MATLAB (MathWorks, United States) and SPSS (version 26, IBM Corp., United States). We extracted the data preprocessing functions from the open-source toolbox HOMER2 to write the data analysis script and used the MATLAB self-contained toolbox SVM in the classification process. The following steps were executed:

##### Preprocessing

A common average reference (CAR) spatial filtering approach was used to reduce global influences and task-evoked physiological noise. The mean of all channels was calculated and subtracted from each single channel for each time point (Bauernfeind et al., 2014). To minimize physiological noises such as heartbeat (1–1.5 Hz) and respiration (0.2–0.5 Hz), the signals were low-pass filtered using the Butterworth fourth-order filter at a cutoff frequency of 0.2 Hz. Additionally, a 0.03-Hz high-pass Butterworth filter of order 4 was used to remove baseline drifts (Scholkmann et al., 2014). Then, data were segmented in 10-s windows from the stimulus onset for further processing.

For statistical analyses, the 20 channels were divided into ROIs which limited the need for multiple statistical comparisons and gave a more simplified overview. We combined neighboring channels which hold the same anatomical locations and similar grand average waveform patterns present in the oxy-Hb response, generating 10 ROIs for the whole cortex we covered in total, as shown in Table 1. For each ROI, two or four neighboring channels with similar waveform patterns in oxy-Hb were averaged.

##### Feature Extraction

We used different time windows to extract candidate features since task-related hemodynamic responses appear with a varying delay of 3–8 s (Bauernfeind et al., 2011). The analysis time period was segregated for 14 parts for feature calculation, consisting of a 2-s time window of 2–4, 3–5, 4–6, 5–7, 6–8, 7–9, and 8–10 s; a 3-s time window of 4–7, 5–8, 6–9, and 7–10 s; and a 4-s time window of 4–8, 5–9, and 6–10 s. Then, the temporal features of fNIRS signals [oxy-Hb], including mean, variance, skewness, kurtosis, and slope values, were independently evaluated for all different time windows, 20 channels to create a candidate-feature pool (Noori et al., 2017).

##### Feature Selection

For each two-class problem, there were a large number of features causing overfitting of a classifier constructed from the training data. In this study, we used the fisher criterion for the feature selection (Power et al., 2011; Moghimi et al., 2012; Hwang et al., 2014). The fisher score based on the Fisher criterion was computed *via*


$$FS_{k}=\frac{{\left(\mu_{i=1}-\mu_{i=2}\right)}^{2}}{\sigma_{i=1}+\sigma_{i=2}}$$

where μ and σ are the mean and variance, respectively, of the designated class *i*. The subscript k represents the *k*th feature element. Since a higher Fisher score signifies larger separability between different classes, the best feature subset was generally constructed by selecting the top *j* feature sets of dimension dim = 1 through dim = 20 we considered.

##### Classification

We evaluated the performance of each subject and the ability to discriminate between their response of different states using a linear support vector machine (SVM) with the leave-one-out cross-validation (LOOCV) method which was commonly used to classify hemodynamic response (Noori et al., 2017; Hosni et al., 2020). SVM has been applied to binary distinction problems for brain machine interfaces (BMIs) and is also widely used for fNIRS signal analysis. In this study, we used SVM to classify oxy-Hb waveforms into different attention-of-direction trials. LOOCV involves one fold per observation (each observation by itself plays the role of the validation set). The (N-1) observations play the role of the training set, and refitting of the model can be avoided. The classification accuracy and mean percentage of observations correctly classified of the 20 repeated model fittings were then calculated and taken as the result.

## Results

This study aimed to examine the cerebral representation in the prefrontal and auditory cortices during sound localization tasks *via* fNIRS. We extracted two sets of fNIRS data for analysis based on behavioral results, used a dichotomous classification method to differentiate the fNIRS signals in different conditions, and presented them in the form of figure legends, which are presented below as part of the results of this experiment.

### Localization Performance

To determine the performance of the subject’s sound source localization in this experimental setup, we recorded the subject’s localization feedback and evaluated it in terms of root mean square error. In addition, we illustrated the specific behavioral performance of all subjects by drawing bubble diagrams.

The RMS results of all participants for different levels are shown in Table 2. Normal-hearing subjects had good sound source localization with root mean square errors in the range of 0°–12°. There were 16 subjects with a 0° RMS and nine with a clear bias. Target–response relationships in two sound levels are depicted in Figure 1F, illustrating the main behavioral results of the study. Sixteen subjects exhibited perfect performance with an accuracy of 100%; some subjects (*n* = 9/25) failed to identify the sound source at ±90°, with mainly −90° being identified as −60° and +90° as +60°. All subjects except two were 100% accurate for 0° and ±30° discrimination. Specifically, S14 mistook sound source 0° as −30° one time, and S19 mistook sound source 0° as 90° one time. We accepted this error and assumed that the participants could successfully identify sound sources from 0° and ±30°. For stimulus levels, there were no statistically significant differences in RMS results between 48 dB and 58 dB (Wilcoxon signed-rank test, *p* = 0.865 > 0.05).

**TABLE 2:** RMS results of low intensity, high intensity, and all trials.

Subject	RMS (low intensity)	RMS (high intensity)	RMS (all trials)
S1*	0	0	0
S3	6	0	4.24
S11	11.23	11.23	11.23
S14	0	4.24	3
S15	4.24	0	3
S17	6	12.73	9.95
S19	15.30	4.24	11.22
S21	10.39	10.39	10.39
S22	7.35	10.39	8.49
S24	0	4.24	3

*The RMS result of S2, S4, S5, S6, S7, S8, S9, S10, S12, S13, S16, S18, S20, S23, S25 is same as S1.

In conclusion, all participants had good performance in the sound localization task in our experimental apparatus.

### Functional Near-Infrared Spectroscopy Results

In this study, to simplify calculation and analysis, we extracted fNIRS data in response to the two questions to be addressed. (1) Does fNIRS detect differences in cortical representations of human attention to different sound source directions between −90°, 0°, and +90°, and if so, (2) are there differences in cortical representations for sound source orientations between −30°, 0°, and +30°?

#### Cortex Representation of −90°, 0°, and +90° Conditions

The feature values of the relative value change of oxy-Hb of all subjects were calculated using a dichotomous method. The details of the classification are listed below:

For high intensity: (1) −90° versus 0°, (2) 0° versus +90°, (3) −90° versus +90°.

For low intensity: (1) −90° versus 0°, (2) 0° versus +90°, (3) −90° versus +90°.

For the same loudspeaker: (1) high intensity versus low intensity for −90°, (2) high intensity versus low intensity for 0°, and (3) high intensity versus low intensity for +90°.

For ipsilateral and contralateral neural ascending: (1) ipsilateral hemisphere: ROIs of the LH for −90° versus ROIs of the right hemisphere for +90° and (2) contralateral hemisphere: ROIs of the right hemisphere for −90° versus ROIs of the LH for +90.

##### Lateral and Front Conditions

Table 3 shows the best classification accuracies of each subject for oxy-Hb responses related to six conditions, including stimuli from −90°/0° in 48 dB, 0°/+90° in 48 dB, −90°/+90° in 48 dB, −90°/0° in 58 dB, 0°/+90° in 58 dB, and −90°/+90° in 58 dB. Most of the subjects (*n* = 18/25, 21/25, 23/25, 21/25, 21/25, 19/25, respectively) showed significantly higher classification accuracies than the marginal classification accuracy of 70%. The mean classification accuracies of the oxy-Hb features were 70.60, 73.60, 78.60, 73.60, 75.60, and 77.40%, respectively. Although the classification accuracies of high intensity were higher than those of low intensity, there was no significant difference between sound level conditions (one-way ANOVA: −90°/0°: *p* = 0.091 > 0.05; 0°/+90°: *p* = 0.114 > 0.05; −90°/+90°, *p* = 0.694 > 0.05).

**TABLE 3:** Classification accuracies of each participant using an optimal selected feature set for oxy-Hb response (–90°/0°/+90°).

	S1 %	S2 %	S3 %	S4 %	S5 %	S6 %	S7 %	S8 %	S9 %	S10 %	S11 %	S12 %	S13 %
48 dB	–90°/0°	80.00	70.00	70.00	75.00	70.00	60.00	75.00	80.00	70.00	60.00	75.00	65.00	75.00
	0°/+90°	70.00	80.00	65.00	70.00	75.00	75.00	70.00	70.00	70.00	70.00	75.00	60.00	75.00
	–90°/+90°	85.00	70.00	85.00	85.00	85.00	80.00	75.00	65.00	90.00	70.00	70.00	80.00	60.00
58 dB	–90°/0°	65.00	85.00	75.00	65.00	65.00	75.00	65.00	85.00	85.00	70.00	70.00	70.00	70.00
	0°/+90°	65.00	85.00	75.00	65.00	85.00	65.00	75.00	70.00	75.00	75.00	70.00	70.00	85.00
	–90°/+90°	70.00	55.00	75.00	85.00	95.00	85.00	90.00	75.00	65.00	75.00	90.00	65.00	80.00

	**S14 %**	**S15 %**	**S16 %**	**S17 %**	**S18 %**	**S19 %**	**S20 %**	**S21 %**	**S22 %**	**S23 %**	**S24 %**	**S25 %**	**Mean**

48 dB	–90°/0°	75.00	80.00	65.00	70.00	75.00	70.00	65.00	60.00	75.00	70.00	70.00	65.00	70.60
	0°/+90°	80.00	75.00	65.00	75.00	80.00	85.00	75.00	75.00	90.00	85.00	70.00	60.00	73.60
	–90°/+90°	70.00	80.00	80.00	80.00	70.00	85.00	100.00	70.00	90.00	90.00	80.00	70.00	78.60
58 dB	–90°/0°	75.00	70.00	70.00	70.00	85.00	70.00	80.00	75.00	80.00	80.00	70.00	70.00	73.60
	0°/+90°	85.00	70.00	80.00	85.00	80.00	75.00	75.00	75.00	80.00	90.00	70.00	65.00	75.60
	–90°/+90°	75.00	65.00	65.00	90.00	80.00	85.00	100.00	60.00	95.00	75.00	70.00	70.00	77.40

**FIGURE 2:**
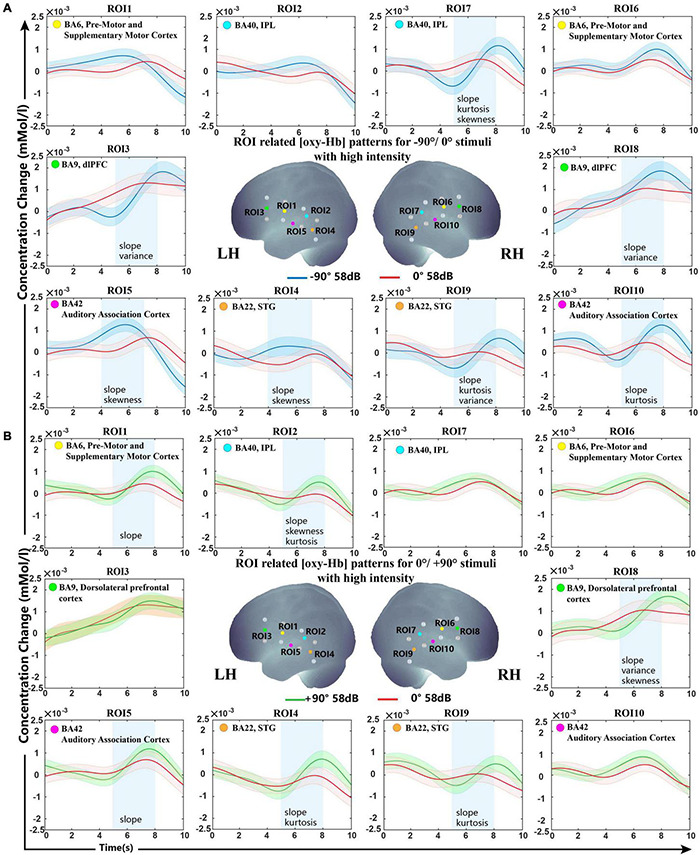
Grand-averaged [oxy-Hb] responses recorded from different locations in high sound intensity (58 dB) for all ROIs and the optimal selected feature set. **(A)** Sound sources from −90° and 0°. **(B)** Sound sources from +90° and 0°. The stimuli were presented at 0 s, and all subjects started concentrating on the sound source. The anatomical location diagram of 10 ROIs is shown in the center of the figure. The lines in blue, red, and green represent the sound sources from −90°/0°/+90°. The selected ROIs with optimal analysis time periods (blue rectangles) and features (shown on the bottom of the blue rectangle) are presented. The shaded regions indicate the standard errors computed across all subjects for the relative condition.

The grand oxy-Hb responses averaged over all subjects are shown in Figure 2, with the best feature set of lateral and front classification with optimal analysis time periods. As shown in the figure, the optimal feature set of −90°/0° was ROIs 3, 7, 8, 9, 10 in 5−8 s and ROIs 4 and 5 in 4–8 s, while 0°/+90° was for ROIs 1, 2, 4, 5, 8, and 9 in 5–8 s, indicating that the bilateral non-primary auditory cortex [including Brodmann (BA) 42 auditory-associated cortex, BA22 STG, and BA 40 Wernicke’s area] executed more use of oxy-Hb for a lateral sound source. For stimuli from −90°, we observed a steeper increase of oxy-Hb in the bilateral BA22, BA42, and BA40 regions of the right hemisphere. For stimuli from +90°, a significant difference was shown in bilateral BA22 and BA42 and BA42 of the LH.

BA9 and BA6 also showed significant differences in our classification. For stimuli from −90°, steeper activation patterns were found in the bilateral BA9. For stimuli from +90°, a significant difference was shown in BA9 of the right hemisphere and BA6 of the LH.

**FIGURE 3:**
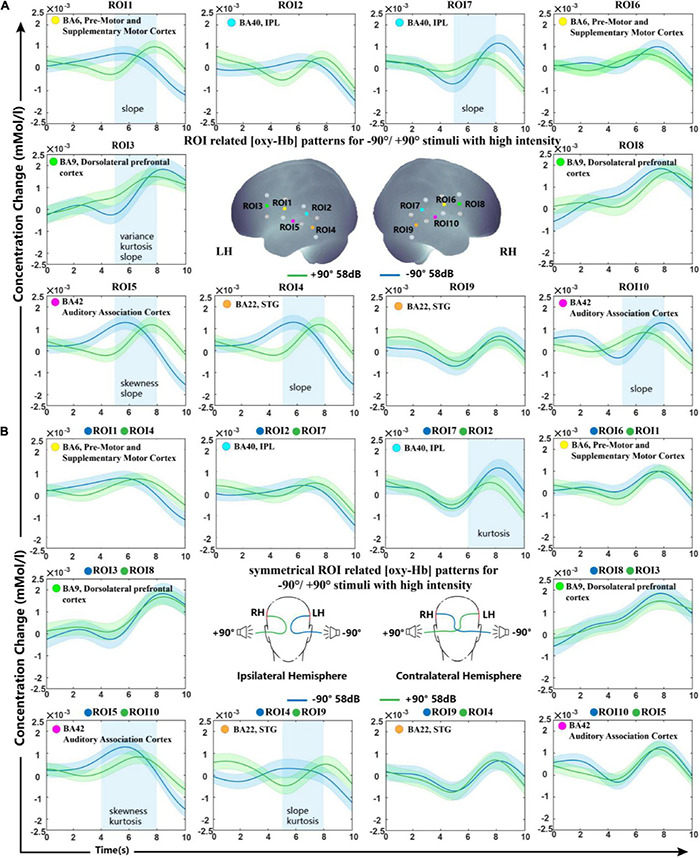
Grand-average oxy-Hb response recorded for –90° and +90° sound sources for all ROIs. **(A)** ROIs in the right and left hemisphere. **(B)** Ipsilateral and contralateral ROI signals of the two conditions. The blue squares on the panel represent time periods in which significant differences in oxy-Hb responses between signals of symmetrical hemisphere ROIs take place. The shaded regions indicate the standard errors computed across all subjects for the relative condition.

Oxy-Hb change waveform patterns of stimuli from −90° and +90° are shown in Figure 3A. Significant differences were observed in BA6, BA9, BA22, and BA42 of the LH and in BA40 and BA42 of the right hemisphere.

Despite the distinct feature type shown in different ROIs, slope is the most frequently selected feature type during the whole LOOCV steps over all subjects. As the feature set results of the two sound levels were not much different, we only presented the high sound intensity in Figure 2. (Low-intensity results are shown in Supplementary Figure S1).

##### Interhemisphere Analysis

In this study, we investigated the difference in spatial tuning between ROIs in the hemisphere ipsilateral and contralateral to the stimulated ear. In comparing the modulation of sound localization cues at the interhemisphere level, the processed signals of symmetrical hemisphere ROIs on stimuli presented from −90° and +90° were then classified using SVM. Figure 3B shows the grand-average oxy-Hb response recorded for all subjects with standard errors. Our statistical analysis indicated significant differences in oxy-Hb changes in the contralateral brain region BA40 and ipsilateral brain regions BA42 and BA22 to the stimulated ear. Grand-average oxy-Hb response showed that the waveform from the −90° source reached its peak 2−3 s earlier than that from the +90° source in both BA42 and BA22 ipsilateral to the stimuli. The kurtosis between the two conditions showed a significant difference in BA40 contralateral to the stimuli during the time period of 6–10 s.

##### Sound Level Conditions

**FIGURE 4:**
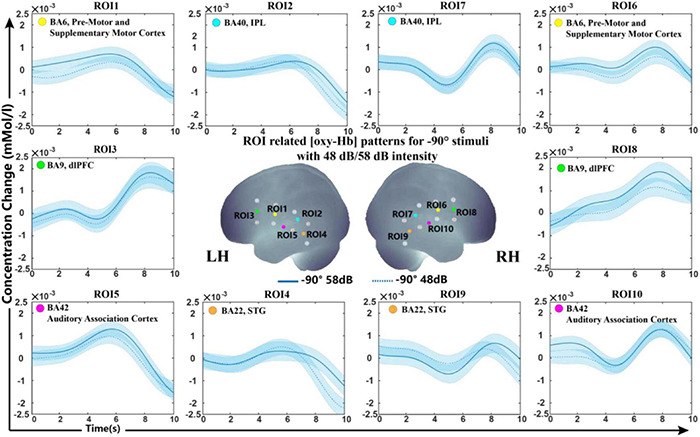
Grand-average oxy-Hb response recorded between different sound levels in the same sound source for 10 ROIs. The blue line in solid and dashed lines on the panel represent oxy-Hb responses between high and low intensities at –90°. The shaded regions indicate the standard errors computed across all subjects for the relative condition.

For the sound level, we further calculated the data to clarify whether this influencing factor affects the results in our experimental setup. Figure 4 shows the grand-average oxy-Hb response recorded between two sound levels (48 and 58 dB) in the −90° sound source for 10 ROIs (the results for 0° and +90° are shown in Supplementary Figure S2). The classification accuracies of the oxy-Hb features on each ROI with five feature types were counted. The average classification accuracy of all subjects in each ROI was lower than 70% (50.81 ± 4.63%). As seen in Figure 4, the grand average oxy-Hb change showed a similar waveform at the two sound levels, indicating that the cortical representation of high intensity was not much different than that of low intensity.

#### Cortex Representation of −30°, 0°, and +30° Conditions

To investigate whether there were significant differences in the cortical representation of −30°/0°/30° sound sources, we further calculated the classification accuracy at high and low sound intensities for sound location of −30° and 0°, 0° and 30°, and −30° and 30° using the optimally selected feature set in −90°/0°/+90°.

The classification accuracy statistics for all subjects are shown in Table 4. The average accuracy for all six classification questions was below 70%, specifically, 66.60, 68.00, 60.00, 68.60, 65.60, and 63.80% corresponding to −30°/0° (48 dB), 0°/+30° (48 dB), −30°/+30° (48 dB), −30°/0° (58 dB), 0°/+30° (58 dB), and −30°/+30° (58 dB), which verified that there were no significant differences in a 30-degrees-of-sound location change on average.

**TABLE 4:** Classification accuracies of each participant using an optimal selected feature set for oxy-Hb response (–30°/0°/+30°).

	S1 %	S2 %	S3 %	S4 %	S5 %	S6 %	S7 %	S8 %	S9 %	S10 %	S11 %	S12 %	S13 %
48 dB	–30°/0°	65.00	70.00	80.00	65.00	80.00	70.00	80.00	60.00	80.00	75.00	60.00	70.00	65.00
	0°/+30°	75.00	60.00	60.00	65.00	35.00	45.00	70.00	70.00	70.00	85.00	60.00	70.00	80.00
	–30°/+30°	65.00	70.00	60.00	60.00	80.00	0.00	70.00	65.00	65.00	65.00	60.00	70.00	60.00
58 dB	–30°/0°	65.00	60.00	65.00	75.00	80.00	75.00	80.00	60.00	65.00	65.00	65.00	60.00	65.00
	0°/+30°	70.00	80.00	50.00	65.00	70.00	65.00	65.00	65.00	55.00	70.00	70.00	70.00	80.00
	–30°/+30°	60.00	65.00	65.00	0.00	65.00	60.00	40.00	60.00	80.00	60.00	80.00	80.00	60.00

		**S14 %**	**S15 %**	**S16 %**	**S17 %**	**S18 %**	**S19 %**	**S20 %**	**S21 %**	**S22 %**	**S23 %**	**S24 %**	**S25 %**	**Mean**

48 dB	–30°/0°	70.00	65.00	25.00	65.00	40.00	65.00	70.00	70.00	65.00	70.00	70.00	70.00	66.60
	0°/+30°	70.00	90.00	75.00	70.00	65.00	80.00	60.00	65.00	80.00	65.00	65.00	70.00	68.00
	–30°/+30°	65.00	60.00	5.00	70.00	75.00	60.00	50.00	70.00	70.00	80.00	60.00	45.00	60.00
58 dB	–30°/0°	70.00	65.00	70.00	75.00	75.00	65.00	60.00	75.00	70.00	65.00	70.00	75.00	68.60
	0°/+30°	65.00	75.00	70.00	40.00	35.00	85.00	65.00	60.00	70.00	65.00	75.00	60.00	65.60
	–30°/+30°	70.00	80.00	65.00	70.00	65.00	75.00	65.00	65.00	80.00	65.00	60.00	60.00	63.80

**FIGURE 5:**
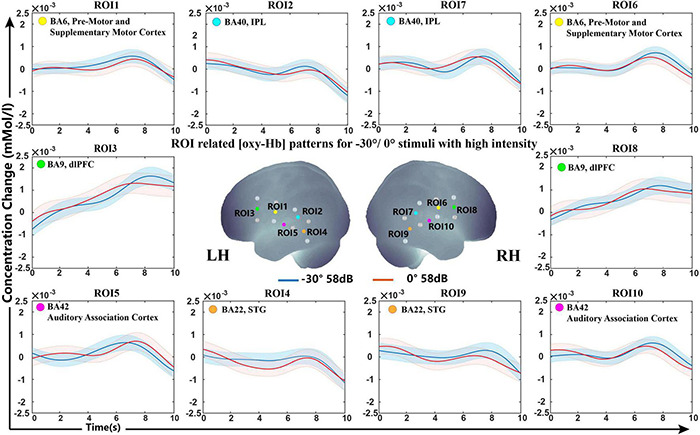
Grand-averaged [oxy-Hb] responses recorded from different locations (–30°/0°) in high sound intensity (58 dB) for all ROIs and the optimally selected feature set.

Grand average concentration change data for sound locations of −30°/0° are shown in Figure 5. The grand-averaged oxy-Hb showed similar waveforms under the −30° and 0° conditions.

## Discussion

This study aimed to investigate what difference the cortical representation was in different sound source localization tasks *via* fNIRS. Differentiation in brain responses related to sound location in 25 subjects was observed. Our experimental evaluation indicated that the lateral and front sound sources revealed different neural activity patterns in the AC and dlPFC.

Many spatial auditory paradigms have proven successful for fMRI and EEG studies (Ebisawa et al., 2011; McLaughlin et al., 2016; van der Heijden et al., 2018). Previous studies using these auditory paradigms identified cortical activation with different sound sources. The block design was generated by referencing the fNIRS and psychophysical experimental methodology. The presented results show a changing amplitude of the oxy-Hb response to the various auditory stimulus sound sources, confirming the feasibility of our experimental design.

### Classification Accuracy and Feature Set

Our behavioral data showed that all subjects had 100% accuracy in identifying the 0° source, while some of them had confusion in −90° and +90°. Although the total trial RMS ranged from 0° to 12°, showing the existence of individual variation in psychophysical performance, our interpretation tended to base on the differences in the mental state between individuals. As shown in Figure 1F, the subjects show a good performance in our sound localization task.

The SVM classification accuracy pointed to the non-primary auditory cortex, including Wernicke’s area and STG, as the structures that showed significant variation in oxy-Hb contribution with different sound sources. The results demonstrated that the fNIRS response to lateral and front sound could be classified with a mean classification accuracy higher than the acceptable practical standard (>70%). As shown in Figure 5, both −90° and +90° sound sources brought about a steep slope and revealed an increased oxy-Hb response during 5−8 s over the STG and part of Wernicke’s area in the contralateral hemisphere, while signals for sound from the front presented a flat curve. Over all of the feature sets selected from LOOCV, the slope showed statistically higher frequencies selected among five different feature types, which indicated that the growth rate of oxy-Hb in different sound sources may characterize the conditions.

Interestingly, a similar result has been shown over the dlPFC in the hemisphere ipsilateral to stimulus sources. The dlPFC is a region most typically associated with higher-level cognitive functions, including working memory and selective attention (Fehr and Krajbich, 2014; Sturm et al., 2016). A previous study indicated that it is possible to causally influence subjects’ choices by making them less likely to express social preferences by disrupting this region. Recently, a high-quality article reviewed and integrated the latest insights from neurophysiological, neuroimaging, and computational modeling studies of mammalian spatial hearing. They proposed that the cortical representation of sound location emerges from recurrent processing taking place in a dynamic, adaptive network of early (primary) and higher-order (posterior-dorsal and dorsolateral prefrontal) auditory regions (van der Heijden et al., 2019). In our research, different neural activities were observed in the dlPFC in both hemispheres between −90°, 0°, and +90°, providing evidence that this region may be involved in the human selection of sound source attention.

### Cortical Correlations of Sound Level

Several research groups have investigated cortical responses to auditory stimuli presented with different sound intensities using different recording techniques (Neuner et al., 2014). Researchers have found different sound intensity modulations of cortical responses to binaural stimuli in the middle and lateral primary auditory cortices, and a linear increase in BOLD signals has been shown in fMRI studies (Uppenkamp and Rohl, 2014). Additionally, the volume of the brain-activated area has been confirmed to be positively correlated with the stimulus level (Rohl and Uppenkamp, 2012). Since the signals measured by fNIRS strongly correlate with the fMRI BOLD signal, the same findings were also found in studies that combined the use of fNIRS (Langers et al., 2007; Behler and Uppenkamp, 2016; Bauernfeind et al., 2018; Weder et al., 2020). It has been shown that the channels overlying the supramarginal and caudal STG evoked a phasic response, and the antero-STG and Broca’s areas showed a broad tonic pattern, where a significant effect of sound intensity level can be observed in early and late time windows, respectively (Weder et al., 2018).

In a previous study, we were interested in sound localization cues rather than sound intensity because we used different sound levels to reduce the monaural effect and check the conformances of oxy-Hb signal shape in the same sound location. As sound intensities at different levels were applied in previous studies from 0 to 100 dB SPL with a gap of approximately 20–30 dB SPL and an analysis time period from onset to 30 s, the results established before may not have been visible in our studies. The sound intensities presented in this study were 48 and 58 dB. The classification accuracy of the oxyhemoglobin waveforms with different sound intensities is below 70%. We considered that such a small difference between sound levels resulted in a marginal difference in waveforms *via* fNIRS. However, it can be observed from the waveform plots that the average waveform peak at 58 dB is above 48 dB in a tiny degree; we conjecture that if we increase the stimulus sound intensity difference, the significant difference between waveforms of sound level will be observed. This might explain why we did not see differences in the cortex of sound intensity in our study.

### Limitation of the Study

fNIRS recordings of auditory stimulation are challenging due to the limitation of time resolution and the limited region we targeted. Since it measures neuronal activity indirectly *via* hemodynamic response, the time resolution reaches the second state, which is a hundred times that in EEG. Previous fMRI studies showed that the initial bilateral transient signal subserved rapid sound detection and that the subsequent lateralized sustained signal subserved detailed sound characterization (Lehmann et al., 2007), which indicated that the time range we analyzed may affect the conclusion we obtained. Moreover, in our study, we used only eight sources and eight detectors fNIRS systems. The cerebral region we covered was limited, and whether there were other region participants in the sound localization activity was unknown. Researchers had applied many stimulus sounds in sound source localization tasks, including pure tones, broadband noise, bandpass noise, and speech sounds. Our study used pink noise in the hope of excluding the effect of speech comprehension on sound source direction recognition in cortical representation. In subsequent studies, we will use speech sound stimuli in complex environments to explore the cortical representation of sound source recognition from the perspective of everyday life applications.

While the best discrimination rate in the same classification among all subjects was 90–95%, the worst was 60–65%. For each subject, the classification accuracies varied in different classifications, as shown in Table 3 (accuracy of 65% at −90° and 0° and 90% at 0° and +90° at high intensities for S4), suggesting an individual variation in our classification models. Since there are many features that may affect the classification, further development of data preprocessing and algorithms needs to be optimized to make the system more flexible. It has been well documented that [deoxy-Hb] responses appear to be more localized and topographically closer to activated areas (Kaiser et al., 2014). Therefore, we plan to search for an effective channel network that combines both oxy-Hb and deoxy-Hb for discrimination to achieve a high-performance system, perhaps *via* classification methods other than SVM.

Although we observed different oxy-Hb response patterns in the non-primary auditory cortex for sound localization, whether this representation is the fusion of bilateral loudness perception and spatial sound perception is not clear. Recent research has shown that non-primary ACs are the regions involved in many auditory perceptions, including horizontal sound direction changes, movement, intensity-independent distance cues, and separation of multiple sound sources (Ahveninen et al., 2014). It is a main concern to provide an effective experimental paradigm for further investigation in spatial cue encoding and functional specialization. Some scientists pointed that future research needed experimental setups using real-life, complex sounds in ecologically valid listening scenes to gain a better understanding of the full complexity of cortical sound location processing. Since sound source localization is a high-order processing and visual information is involved in an important part of the sound localization activity, a dark room environment was used in this experiment to avoid the extent of visual information input. The inclusion of visual information stimuli and attention to the visual, audiovisual cortex is needed in subsequent studies.

Numerous studies have concentrated on brain function in transmitting and tuning binaural sound localization cues by means of neurophysiological methods, EEG, MEG, and fMRI over the past decade (Zatorre et al., 2004; Krumbholz et al., 2005; Palomaki et al., 2005). In general, our study only investigated the availability of fNIRS on sound localization irrespective of whether ITD and/or ILD were used to produce spatial perception. Although we observed the cortical representation of different spatial origins of sounds, it is not known when distinguishing the location of sound sources to what degree the binaural spatial cue weighs in our recorded fNIRS signals in the revolved cortex. Besides, the sound level to the ipsilateral eardrum would be increased when a sound source is moved from frontal position to lateral position because of the sound scattering characteristics of the head and ear (Liang and Yu, 2020; Liang et al., 2021). Since none of the alternative methods completely eliminates monaural level cues, the available neuroimaging studies of ILD processing all have monaural cues as a potential confound (Ahveninen et al., 2014). Therefore, further comparison and integration of studies are needed to obtain cortical activity representations of separate spatial cues from sound sources.

### Outlook

Our study shows that fNIRS is a valid and reliable assessment tool for sound localization task-associated oxygenated blood. However, we know that in real life, sound source localization activities are not just about the directional recognition of simple sounds. Multidirectional speech perception in noisy environments and competing speech sounds are also spatial sound source perceptions. We speculate that future research on spatial acoustic brain function will be devoted to a better understanding of sound source localization processing in real-life complex speech environments.

The application of fNIRS to investigate cross-modal plasticity and speech processing has been of interest in disabled groups like cochlear implantation (CI) users in recent years, from which we have identified the potential of speech development among children early and timely intervened for treatment. As the increasing number of binaural cochlear implant users results in more demands, such as better speech perception in noise and sound localization ability, understanding the cortical representation differences between normal people and guiding and assessing the fitting of bimodal or bilateral CI have been new issues. Due to the previous fNIRS contributions in speech perception, we hold the opinion that the cortical perception of spatial speech sounds will be carried out well off.

We believe that fNIRS holds great potential for growth and application in the clinic, offering new possibilities for the functional organization of the brain in the spatial auditory field.

## Conclusion

This study presented an experimental paradigm for measuring the cortical representation of sound localization under sound fields *via* fNIRS. We investigated the differences in cortical representations of different sound sources during listening tasks *via* fNIRS. The main waveform patterns of oxy-Hb demonstrated that the front and lateral sound sources extracted different neural activity in non-primary AC and dlPFC.

Taken together, our findings suggested that fNIRS could detect differences in cortical representations of sound source directions from the lateral and the front, providing evidence for the cerebral activation patterns of spatial hearing.

## Data Availability Statement

The original contributions presented in the study are included in the article/Supplementary Material, further inquiries can be directed to the corresponding author/s.

## Ethics Statement

The studies involving human participants were reviewed and approved by Human Subjects Committee of the Southern Medical University. The patients/participants provided their written informed consent to participate in this study.

## Author Contributions

HZ and FC designed the research. XT performed the research and wrote the first draft of the manuscript. XT and ZG analyzed the data. XT, YL, JC, HZ, FC, and JT edited the manuscript. XT, YL, JC, and HZ wrote the manuscript. All authors contributed to the article and approved the submitted version.

## Conflict of Interest

The authors declare that the research was conducted in the absence of any commercial or financial relationships that could be construed as a potential conflict of interest.

## Publisher’s Note

All claims expressed in this article are solely those of the authors and do not necessarily represent those of their affiliated organizations, or those of the publisher, the editors and the reviewers. Any product that may be evaluated in this article, or claim that may be made by its manufacturer, is not guaranteed or endorsed by the publisher.
