# Supplementary material for: Cerebral Representation of Sound Localization Using Functional Near-Infrared Spectroscopy
Source: Front Neurosci. 2021 Dec 14;15:739706. doi: 10.3389/fnins.2021.739706 (PMC8712652; doi:10.3389/fnins.2021.739706)
Supplement: Supplementary file 1 [file Image_1.pdf]

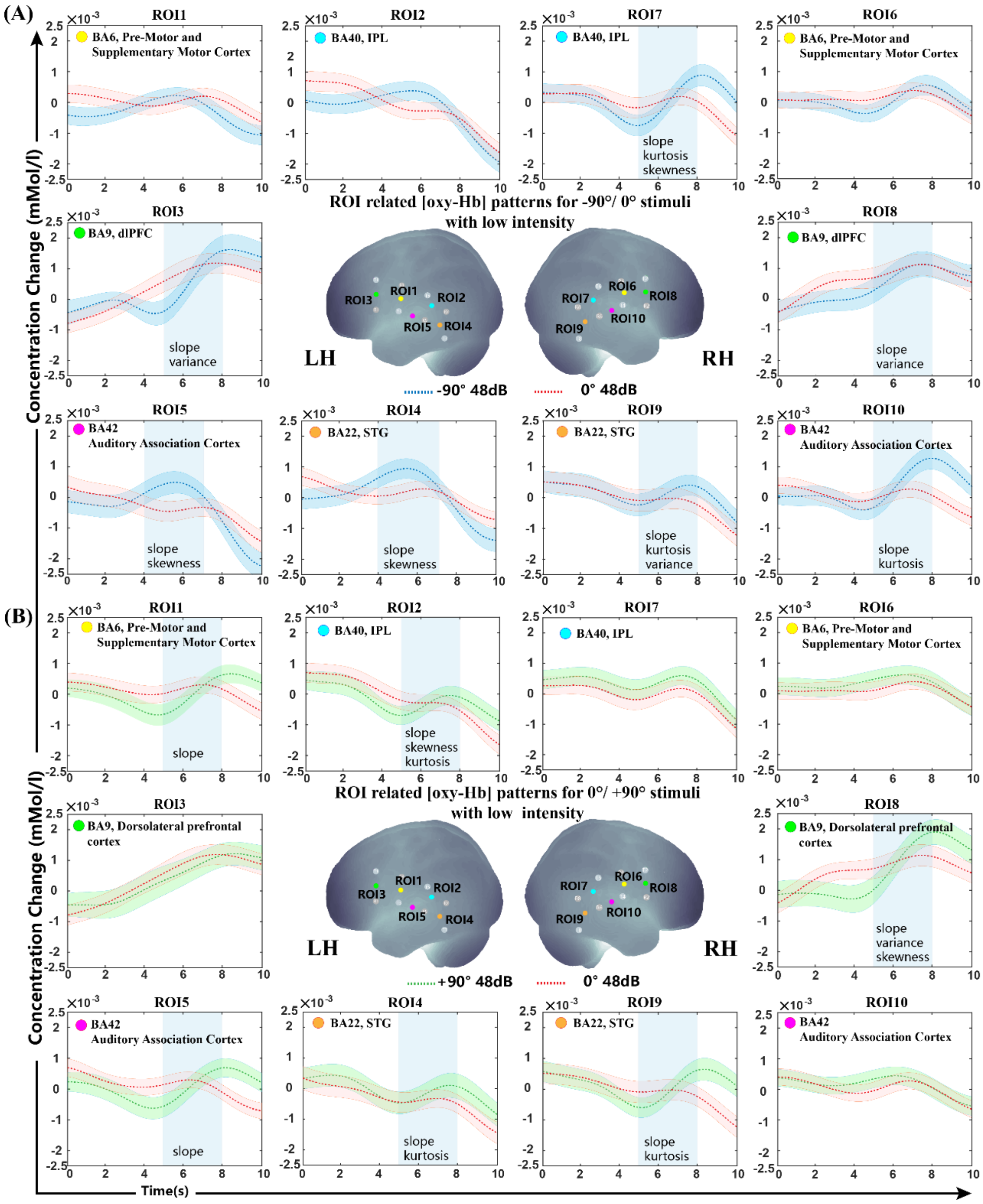

Figure S1 Grand-averaged [oxy-Hb] responses recorded from different locations (-90°/0°/+90°) in low sound intensity (48 dB) for all ROIs and the optimal selected feature set. (A) sound sources from -90° and 0°. (B) sound sources from +90° and 0°. The stimuli were presented at 0 s, and all subjects started concentrating on the sound source. The anatomical location diagram of 10 ROIs is shown in the center of the figure. The lines in blue, red, and green represent the sound sources from -90°/0°/+90°. The selected ROIs with optimal analysis time periods (blue rectangles) and features (shown on the bottom of the blue rectangle) are presented. The shaded regions indicate the standard errors computed across all subjects for the relative condition.
