# Supplementary material for: Cerebral Representation of Sound Localization Using Functional Near-Infrared Spectroscopy
Source: Front Neurosci. 2021 Dec 14;15:739706. doi: 10.3389/fnins.2021.739706 (PMC8712652; doi:10.3389/fnins.2021.739706)
Supplement: Supplementary file 2 [file Image_2.pdf]

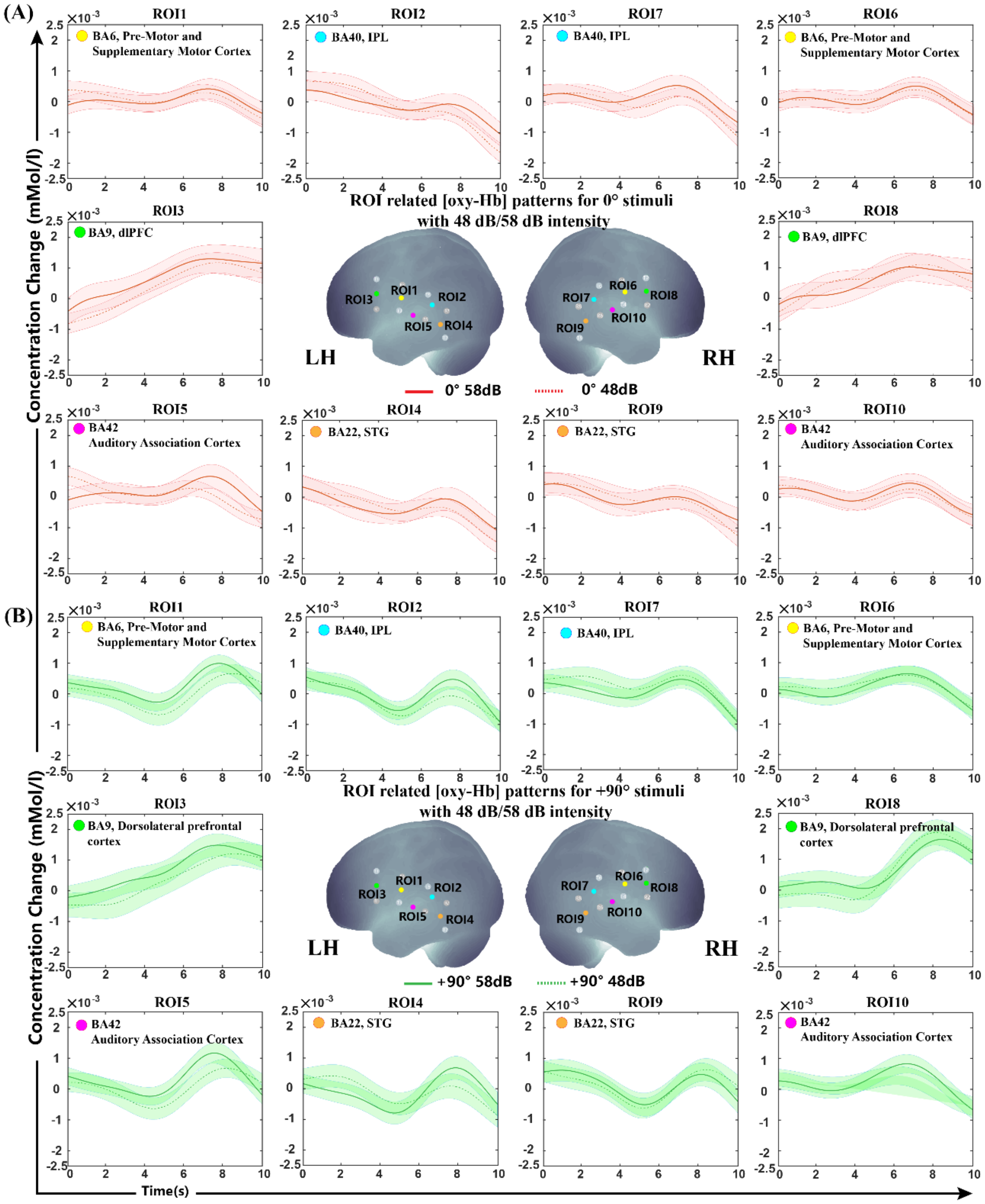

Figure S2 grand-average oxy-Hb response recorded between different sound levels in same sound source for 10 ROIs. The red and green line in solid and dashed on the panel represent oxy-Hb responses between high, low intensities in (A) 0° and (B) +90°. The shaded regions indicate the standard errors computed across all subjects for relative condition.
